# Metabolic Equivalent and its Associated Factors in a Rural Community of Karnataka, India

**DOI:** 10.7759/cureus.4974

**Published:** 2019-06-22

**Authors:** Shalini Chandrashekar Nooyi, Srinivasa Nandagudi Murthy, Shalini Sivananjiah, Pruthvish Sreekantaiah, Dinesh Rajaram, Vanitha Gowda, Krishnamurthy Ugraiah

**Affiliations:** 1 Community Medicine, M. S. Ramaiah Medical College, Bengaluru, IND; 2 Non-Communicable Diseases, Indian Council of Medical Research - National Centre for Disease Informatics and Research, Bengaluru, IND; 3 Biochemistry, M. S. Ramaiah Medical College, Bengaluru, IND

**Keywords:** mets, metabolic equivalents, physical activity, rural, india

## Abstract

Background

Health benefits of physical activity measured in terms of metabolic equivalent minutes (MET-minutes per week) have been established. However, factors affecting physical activity, like age, gender, body mass index, waist-hip ratio, particularly in rural communities have not been documented on a large sample.

Methods

Baseline physical activity data of more than 4000 subjects over 30 years of age, who were enrolled in a randomised community-based study on non-communicable diseases, were analysed. Global Physical Activity Questionnaire was used and anthropometric measurements were classified according to the MONICA study manual. Three domains of physical activity were measured as MET-minutes per week - activity at work, travel to and from places and recreational activities. Association of MET-minutes with sociodemographic variables and risk factors for cardiovascular diseases was studied.

Results

Mean MET-minutes per week of females were found to be significantly lower than that of males and decreased with advancing age and higher BMI in both genders. Married persons, normal BMI, normal waist-hip ratio, lower leisure time activity had demonstrated higher MET values (P = 0.000). In our study, the prevalence of inactivity (<600 MET-minutes) was 3.2% which was similar in both males and females. As high as 96.2% of the subjects had MET-minutes of > 1200. Nearly 50% of the subjects had leisure time ranging from 121 to 240 minutes per day.

Conclusion

A large majority of adults over 30 years of age in a rural community in Karnataka (96.2%) had very high MET-minutes per week of >1200 per day. Abnormal BMI, higher waist-hip ratio and more leisure time were associated with lower MET-minutes which are modifiable. About 50% had more than 2 hours of leisure time per day. It is recommended that health promotion for active lifestyle should be encouraged.

## Introduction

Levels of physical inactivity are rising in many countries with major implications for the general health of people worldwide and for the prevalence of non-communicable diseases (NCDs) such as cardiovascular disease, diabetes and cancer and their risk factors such as raised blood pressure, raised blood glucose and overweight. Physical inactivity is estimated as being the principal cause for approximately 21-25% of breast and colon cancer burden, 27% of diabetes and approximately 30% of ischaemic heart disease burden [1]. There is a growing awareness that physical inactivity and lack of physical fitness may be risk factors for several cancers [2].

Globally, about 2 million (5.5%) deaths occur every year and 2.1% of the disability-adjusted life years (DALYs) lost is due to physical inactivity. Further, obesity or overweight causes 2.8 million deaths globally and 2.3% of global DALYs [3]. In May 2004, the 57th World Health Assembly recommended that member states develop national physical activity action plans and policies to increase physical activity levels in their populations [4].

Physical activity is defined by WHO as any bodily movements that require energy expenditure. It includes exercise as well as other activities done as part of playing, working, active transportation, house chores and recreational activities. Physical inactivity (lack of physical activity) is identified as the fourth leading risk factor for global mortality [1]. Insufficient physical activity is one of the four primary risk factors for NCDs [5].

About a decade ago, WHO developed the Global Physical Activity Questionnaire (GPAQ) for physical activity surveillance. Since it has been found to be valid and reliable, and also adaptable to cultural differences, it has been used in more than 100 countries globally. GPAQ measures physical activity in an objective form with the help of metabolic equivalents [6].

Since we do not have adequate information on physical activity measured in terms of metabolic equivalent minutes (MET-minutes/week) in Karnataka, particularly in rural communities, this communication reports the quantum of physical activity and its determinants in a large population. Further, the association between MET-minutes/week with sociodemographic factors and anthropometric measurements have been described.

## Materials and methods

The participants of the present analysis were recruited into a randomized community intervention trial funded by the Indian Council of Medical Research (ICMR) conducted in Kaiwara and Kurubur primary health centre (PHC) areas (situated north-east of Bangalore at a distance of 70 km) of Chintamani Taluk, Chikballapur District, Karnataka, India (unpublished data). Mylapura subcentre area was selected from Kaiwara and Doddaganjur subcentre area was selected from the another PHC area by simple random sampling procedure. For randomization, by tossing a coin, Mylapura was allocated to intervention arm and Doddaganjur as comparison arm. Persons >= 30 years of age were the study participants. For this analysis, the baseline data of the participants of both the arms (intervention and comparison groups) have been combined.

Study population and sample size for the baseline study

Participants who were residents for at least six months in the study area were eligible. Mentally challenged, bedridden or differently abled participants were excluded for this analysis since physical activity information could not be obtained.

A baseline survey was conducted in the year 2013 in both areas, on several parameters including physical activity. We used the GPAQ questionnaire which is part of the WHO STEPs tool. The GPAQ has undergone research and shown to be valid and reliable. It is also adaptable to incorporate cultural and other differences. It has been used in more than 100 countries globally, mainly through the WHO STEPwise approach to NCD risk factor surveillance (STEPS). The GPAQ covers several components of physical activity, such as intensity, duration, and frequency, and it assesses three domains in which physical activity is performed (occupational physical activity, transport-related physical activity, and physical activity during discretionary or leisure time). Complete enumeration of persons ≥30 years in both the arms was done during the baseline survey which revealed that there were 4576 persons in this category. Of these, 4246 (92.8%) adults ≥ 30 years of age fulfilled the inclusion and exclusion criteria were included for this analysis. The sample required for the study was 2100 participants in each arm of the study. Ethical clearance was obtained from the Institutional Ethical Review Board.

Participants were interviewed in their homes by trained medical social workers. Each eligible participant was administered a separate pretested structured interview tool to gather details on sociodemographic, morbidity and risk factors for non-communicable diseases.

Following the interview, trained physicians measured anthropometric measurements: weight, height, waist and hip circumference, using the procedure of the World Health Organization: Multinational MONItoring of trends and determinants in CArdiovascular disease (WHO MONICA) study [7]. Body mass index was classified using cut offs of BMI for Asians [8]. Waist and hip circumference (HC) were classified according to a joint interim statement of international organizations [9].

Data on physical activity during any typical week was collected using the GPAQ questionnaire and analysed according to the GPAQ analysis guide, in which intensity of physical activity, the total time spent on physical activity during a typical week and the number of days were considered and was converted to MET-minutes/week. Three domains of physical activity were measured as MET-minutes/week scores-activity at work, travel to and from places and recreational activities. MET-minutes/week scores were calculated separately for individual domains and sub-domains, adopting existing guidelines of GPAQ and added together to obtain MET-minutes/week score for all domains. When calculating a person's overall energy expenditure using GPAQ data, 4 MET-minutes/week were assigned to the time spent in moderate activities, and 8 MET-minutes/week to the time spent in vigorous activities [10]. Leisure time spent per day was also recorded. Privacy and confidentiality were maintained during data collection. The major advantage of the GPAQ questionnaire is that, it can assess physical activity in each domain separately in addition to the total physical activity and also has been previously validated in nine populations including Asian Indians and found to be reproducible and reliable [11]. For the calculation of a categorical indicator, the total time spent on physical activity during a typical week, the number of days as well as the intensity of physical activity were taken into account. Further in the GPAQ, MET-minutes/week has been classified into three groups as: Inactive/low (<600 MET-minutes/week), active (600-1200 MET-minutes/week) and highly active (>1200 MET-minutes/week).

Statistical analysis

Data was analyzed using the SPSS Version 18.0 (SPSS Inc., Chicago, IL), through frequency distribution tables. Descriptive statistics was employed for continuous variables, while proportions/mean, standard deviation, median and interquartile range were calculated for continuous variables, like MET-minutes/week, age. Mann-Whitney test was used to test the differences in the mean values of MET-minutes/week between groups such as gender, age, waist-hip ratio. Since the data was non-normal. Kruskal-Wallis test followed by post-hoc test was used to test for the differences in mean MET-minutes/week values amongst various groups such as age, BMI etc. Categorical variables/dichotomized variables were expressed as percentages and associations were tested by Chi-squared test of significance.

## Results

The total number of households surveyed were 2361, which accounted for 4246 individuals, of which 1946 were males and 2300 were females.

The prevalence of insufficient physical activity in adults aged 30+ years was found to be only 3.2% (defined as less than 150 minutes of moderate-intensity activity per week which is equivalent to less than 600 MET-minutes/week), and was found to be similar in both genders. The overall mean MET-minutes/week was found to be 11432.8. Males had higher mean MET-minutes/week values as compared to females and the differences in the mean values were found to be statistically significant (P = 0.001). Difference was noted between mean, median and mode values indicating that the distribution of MET-minutes/week was non-normal (Table 1). Hence, in all the further results, it is proposed to provide median MET-minutes/week.

**Table 1:** Distribution of total MET-minutes/week and mean, median and mode by gender MET: Metabolic equivalent

MET-minutes/week	Male	Female	Total
	N (%)	N (%)	N (%)
<600	63 (3.2)	74 (3.2)	137 (3.2)
600-1200	9 (0.5)	74 (0.7)	24 (0.6)
>1200	1874 (96.3)	2211 (96.1)	4085 (96.2)
Total	1946	2300	4246
Mean (SD)	11833.0 (5864.2)	11094.0 (5940.7)	11432.8 (5916.5)
Median (IQR)	12240.0 (7320.0-15120.0)	10560.0 (6480.0-15120.0)	11640.0 (6960.0-15120.0)
Mode	15120.00	15120.00	15120.00

Decreasing value of median MET-minutes/week (13080-3600) with increasing age was observed, which was statistically significant (P = 0.001) (Table 2). Pearson's correlation coefficient was computed between age and MET values in terms of logarithmic transformation, which revealed statistically significant negative correlation (r = -0.273, P = 0.001). It was noted that median MET-minutes/week were the highest in 30-39 years age group when both genders were considered. In males, the median MET-minutes/week were the same in 30-39 and 40-49 years age groups and then decreased with age. In females, a continuous decreasing trend was noted. The differences in the median MET-minutes/week in different age groups were statistically significant (P = 0.000) in both genders and in the total.

**Table 2:** Distribution of MET-minutes/week scores (Median and IQR) by age and gender of subjects MET: Metabolic equivalent; IQR: Interquartile range.

	Male			Female			Total		
Age (Years)	N %	Median	IQR	N %	Median	IQR	N %	Median	IQR
30-39	564 (29.0)	13440	9360-15120	769 (33.4)	13260	8640-15120	1333 (31.4)	13080	9360-15120
40-49	572 (29.4)	13440	9360-15120	642 (27.9)	11280	7200-15120	1213 (28.6)	12240	7890-15120
50-59	349 (17.9)	12240	6960-15120	423 (18.4)	10680	6960-15120	772 (18.2)	11280	6960-15120
60-69	299 (15.4)	10800	6000-15120	336 (14.6)	8880	3360-13440	635 (14.9)	10200	5040-15120
70-79	126 (6.5)	7140	3000-13440	100 (4.3)	6480	2880-10080	226 (5.3)	6720	2880-11760
80 and above	36 (1.8)	3600	720-10080	30 (1.3)	3540	1650-10080	66 (1.5)	3600	1530-10080
Total	1946	12240	7320-15120	2300	10560	6480-15120	4246	11640	6960-15120
Kruskal–Wallis Test	P = 0.000			P = 0.000			P = 0.000		

Table 3 shows the MET-minutes/week distribution by educational and occupational levels of the study population.

**Table 3:** Distribution of MET-minutes/week by education and occupation status of subjects MET: Metabolic equivalent; IQR: Interquartile range.

	Male			Female			Total				
Education	N %	Median	IQR	N %	Median	IQR	N %		Median		IQR
Below graduation	1846 (94.9)	13020	6790-13860	2281 (99.2)	10560	6480-15120	4127 (97.2)		11640		6960-15720
Graduate, professional	100 (5.1)	10140	6720-13860	19 (0.8)	8520	5160-14640	118 (2.8)		10800		6720-13920
Total	1946			2300			4241				
Occupation of subjects											
Unemployed	46 (2.4)	240	120-3360	82 (3.6)	2400	120-6720		128 (3.0)		1560	120-5250
Unskilled, semi-skilled, skilled	556 (28.6)	13440	8280-15600	526 (22.8)	12360	7320-15120		1082 (25.5)		13440	7770-15120
Clerical, shop owner, semi-professional, professional	488 (25.0)	11700	7710-15120	135 (5.9)	9600	5880-15120		623 (14.6)		10920	7320-15120
Housework	-	-	-	701 (30.5)	11760	7680-14640		701 (16.5)		11760	7680-14640
Farmer	856 (44.0)	12000	7400-15120	856 (37.2)	10320	5880-15120		1711 (40.3)		10920	6960-15120
Total	1946	12240	7320-15120	2300	10560	6480-15120		4246		11640	6960-15120

Educational status was dichotomized into two groups - below graduation and above graduation, since participants who did not complete graduation were employed as labourers/farmers.

Education of the participants played a role in the activity levels. Those who were graduates or professionals had lower MET-minutes/week compared to those who had lower levels of education, in both males and females. This corroborates with the fact that, highly educated subjects had sedentary jobs and lifestyles, while the less educated were probably doing agricultural or skilled labour. Median MET-minutes/week was found to be highest in unskilled personnel, while it was lowest among unemployed males.

Table 4 shows that about a quarter of the population were undernourished (BMI < 18.5) and the proportion was higher in females (28.9%) when compared to males (18.8%) which was statistically significant (P = 0.001). Persons with BMI >= 30 were 2.5% and it was almost the same in males and females (P = 0.9). It was observed that higher proportion of females were obese as compared to males and the differences were found to be statistically significant (P = 001).

**Table 4:** Distribution of median MET-minutes/week by BMI of the subjects BMI: Body mass index; MET: Metabolic equivalent; IQR: Interquartile range.

BMI	Male			Female			Total		
	N %	Median	IQR	N %	Median	IQR	N %	Median	IQR
<18.5	360 (18.6)	11760	6870-15120	655 (28.6)	10320	6360-15120	1015 (24.0)	10320	6720-15120
18.5-22.9	986 (50.8)	13440	8580-15120	1077 (47.1)	11760	7320-15120	2063 (48.8)	12000	7440-15120
23.0-29.9	552 (28.5)	11640	7200-15120	491 (21.5)	10200	5880-13680	1043 (24.7)	10320	6600-15120
>=30	42 (2.2)	10320	5250-15540	64 (2.8)	10080	5220-12930	106 (2.5)	10080	5250-13680
Total	1940*	13440	8400-15120	2287*	11760	7200-15120	4227	11760	7920-15120
Kruskal–Wallis Test	P = 0.223			P = 0.001			P = 0.000		
*In six male and 13 female subjects BMI could not be calculated since their height could not be measured.									

Median MET-minutes/week 12000 for both genders combined were found in the group with normal BMI (18.5-22.9) which was the highest in comparison with other categories of BMI. Similar observations were found in both males and females. Lowest MET-minutes/week were found in obese persons in both males and females. The differences in MET-minutes/week among different BMI categories were statistically significant in females and both genders combined (P = 0.001).

Nearly 75% of males and 40% of females had substantially increased risk based on the waist-hip ratio, which was found to be statistically significant (P = 001) (Table 5). In both males and females, participants with substantially increased risk had lower median MET-minutes/week than in those with no risk (P = 0.001).

**Table 5:** Distribution of MET-minutes/week (median and IQR) by waist-hip ratio and gender of subjects MET: Metabolic equivalent; IQR: Interquartile range.

	Male			Female		
Waist-hip ratio	N (%)	Median	IQR	N (%)	Median	IQR
No risk (Male < 0.9, Female < 0.85)	477 (25.5)	13440	10800-15120	1357 (61.9)	12240	8160-15120
Substantially increased risk (Male >= 0.9, female >= 0.85)	1394 (74.5)	11520	6840-15120	834 (38.1)	8760	5280-13440
Total	1871	13440	8400-15120	2191	11760	7200-15120

The average leisure time spent was 2.5 hours in males and females as well as males and females combined (Table 6). As the leisure time increased over 2.5 hours per day, mean MET-minutes/week decreased.

**Table 6:** Distribution of MET-minutes/week (median and IQR) by leisure time of subjects MET: Metabolic equivalent; IQR: Interquartile range.

Leisure time-Minutes per day	Male			Female			Total		
	N %	Median	IQR	N %	Median	IQR	N %	Median	IQR
<=120	1023 (52.5)	10320	6840-15120	989 (43.0)	9600	5880-13680	2012 (47.4)	10200	6000-14640
121-240	893 (45.9)	13440	10080-15120	1249 (54.3)	13440	8640-15120	2142 (50.4)	13440	8880-15120
>241	30 (1.5)	1920	240-5190	62 (2.7)	2040	120-6750	92 (2.2)	2040	120-6720
Total	1946			2300			4246		

## Discussion

Very few studies on physical activity have been carried out in the country employing GPAC questionnaire and have reported the results in terms of MET-minutes/week. The present study is one of the largest to report on physical activity patterns for rural population (n = 4246) from the state of Karnataka, India. A major strength of our study was its large sample size, employing collection of data through validated questionnaire for measuring the physical activity. The study has measured physical activity in terms of MET-minutes/week and examined the relationship between MET-minutes/week with various risk factors of non-communicable diseases in the southern region of Karnataka, India.

In the present study, the mean (SD) and median (IQR) MET-minutes/week was found to be 11432 (SD = 5916.5) and 11640 (IQR = 6960-15120). Only 3.2% of the population had MET-minutes/week below or equal to 600 (physically inactive). A great majority of the population (96.2%) had more than 1200 MET-minutes/week. Males had significantly higher MET-minutes/week compared to females.

According to ICMR-INDIAB study done in 2014, on 14227 individuals, the prevalence of physical inactivity among rural population was 50.0%. Overall, the study estimated that 392 million people in India were inactive [12]. The ICMR-IDSP study has reported that the percentage of population with lower than the WHO recommended levels of physical activity was 42% in Madhya Pradesh, 66% in Tamil Nadu, 68% in Andhra Pradesh, 81% in Maharashtra and 76% in Kerala. These differences may be possibly due to occupational differences in the study samples. In this study also, the proportion of persons with high MET-minutes per week was large, probably since it was an agrarian population. The 51 country study done worldwide and carried out a decade earlier, reveals a high variability in physical activity [13]. The findings of the study showed overall physical inactivity to be much lower (17.7%; 19.8% women and 15.2% men). In the World Health Survey (WHS), the prevalence of physical inactivity in India was 9.3% in men and 15.2% in women. This figure is more or less similar to our findings. However, the WHS used the IPAQ (International Physical Activity Questionnaire), whereas in the present study we have used the GPAQ, which could account for some of the differences seen.

A review of 55 population-based surveys of physical activity from 29 Asia-Pacific countries was published recently [14]. Varying methodologies were used in the different studies - 19 surveys used the IPAQ, 18 surveys used the GPAQ and 18, other instruments. The review demonstrated that physical activity estimates vary widely even within a single country using different surveys in similar time periods. Three surveys from India were included in the above review - the WHO Modified STEPS Survey (GPAQ) in 2003-2005, the World Health Survey (IPAQ) in 2003 and the IPAQ Short Form (2003). The prevalence rates of “sufficiently active” were 84, 88 and 77%, respectively. These figures are similar to the findings of the present study.

Studies in various countries including Asian countries showed that the prevalence of “low physical activity” varied from 9% to 58% [15,16]. A study done by Shah and Mathur, in 2005 in six regions of India (Delhi and Ballabgarh in the north, Chennai and Trivandrum in the south, Nagpur in the west and Dibrugarh in the east) showed that overall inactivity levels were 12.6% in males and 18.9% in females [17]. Moreover, this study also showed marked variability in physical activity in different regions of the country. A study done in rural Vellore in 2012, showed prevalence of physical activity among rural women to be 46.4% with high prevalence of most of the risk factors of NCDs like overweight (34.6%) and central obesity (55.3%) [18]. In the current study also, there was a variability in physical activity associated with different factors.

The present study reports a very high level of physical activity (highly active). This could be possibly due to the fact that the majority of the population was agrarian/skilled workers, in the prime of their life (productive age of 30-59 years). Further, 47% of the subjects spent only <120 minutes per day as leisure time.

There was a significant difference in median METs - between males and females, with males having higher median MET-minutes/week. Our findings are in agreement with other studies which report higher physical activity levels in males (work) compared to females [16,17,19,20]. Globally, 23% of adults aged 18 years or older were insufficiently active (men 20% and women 27%) [21].

A study done in Nepal in a peri-urban community to assess its variations across different sociodemographic correlates, revealed that women and housewives and older, more educated and self- or government-employed respondents showed a greater prevalence of physical inactivity [22]. Similar findings of physical activity levels have been reported in a study conducted in Malaysia [23].

Persons who had higher BMI and higher waist-hip ratio revealed lower MET-minutes/week. Seventy-four percent of males and 38.1% of females had a substantially increased risk of noncommunicable diseases by waist-hip ratio, wherein the median MET-minutes/week were much lower than that of the “no risk” category. Decreasing median MET-minutes/week with increasing BMI levels as reported by us have also been reported by other studies (ICMR-INDIAB) [12]. Physical activity levels and weight of subjects seem to form a vicious cycle and are inversely related. A cross-sectional study conducted in a village in Karnataka State, India, through a house-to-house survey of persons ≥ 18 years of age, employing a questionnaire and laboratory investigations, anthropometric measurements, blood pressure, has revealed a significant association of BMI with gender, age, waist-hip ratio, systolic and diastolic blood pressure, fasting triglycerides (all P = 0.001) and high-density lipoprotein (HDL) (P = 0.038) [24]. Physical activity was assessed in this study and only a small proportion of the people were leading a sedentary lifestyle. It was noted that 61.3% and 48% of males and females were engaged in heavy work or manual labour (unpublished data). Association was found between waist-hip ratio and physical activity in other studies also [25, 26]. Another study showed a significant association between physical activity and BMI especially in obese individuals [27].

Persons doing 150 minutes of moderate physical activity each week (or equivalent) are estimated to have a reduced risk of ischaemic heart disease by approximately 30%, the risk of diabetes by 27% and the risk of breast and colon cancer by 21-25%. Additionally, physical activity lowers the risk of stroke, hypertension, and depression. A greater number of hours of leisure-time may indicate attitudes towards physical activity and lack of awareness about its importance in maintaining BMI.

The Government of India has initiated a national programme for prevention and control of cancer, diabetes, cardiovascular diseases and stroke with the objectives of (a) creating awareness on lifestyle changes, (b) early detection of NCDs and (c) capacity building of health systems to tackle NCDs [28]. In addition, improving opportunities for physical activity in rural areas like walking and cycling may be a cost effective intervention [29].

Limitations

The cross-sectional nature of this study has limited our ability to infer causal association between physical activity and risk factors for NCDs. Additionally, daily activities at home such as cleaning, cooking, washing, child care, and other household activities, were not assessed. There might have been some amount of recall and information bias, since physical activity measures were self-reported, leading to over or underreporting of physical activity.

## Conclusions

Our study contributes to the growing body of evidence that physical activity measured in a more objective manner through MET-minutes/week, will be more helpful for quantifying and associating risk factors for chronic diseases. Various sociodemographic characteristics of subjects, such as age, gender, education, occupation status have also contributed to the associations of MET-minutes/week with various factors. Our study showed decreasing MET-minutes/week with increasing age. Males and unskilled and semiskilled workers had the highest MET minutes per week. Persons with normal BMI, appropriate waist-hip ratio and leisure time of 2-4 hours per day, had higher MET-minutes per week.

Recommendations

In order to reduce the risk of cardiovascular disease which is indicated by the waist-hip ratio and BMI, WHO recommends the following: (a) adults aged 18-64 should do at least 150 minutes of moderate-intensity aerobic physical activity throughout the week or do at least 75 minutes of vigorous-intensity aerobic physical activity throughout the week or an equivalent combination of both, (b) aerobic activity should be performed in bouts of at least 10 minutes duration.

## References

[1] World Health Organization (2009). Global Health Risks: Mortality and Burden of Disease Attributable to Selected Major Risks. https://www.who.int/healthinfo/global_burden_disease/GlobalHealthRisks_report_full.pdf.

[2] Murthy NS, Mukherjee S, Ray G, Ray A (2009). Dietary factors and cancer chemoprevention: an overview of obesity-related malignancies. J Postgrad Med.

[3] (2018). Global health repository. https://www.who.int/gho/ncd/en/.

[4] (2019). Fifty-seventh World Health Assembly, Geneva, 17-22 May 2004: resolutions and decisions, annexes. http://www.who.int/iris/handle/10665/20159.

[5] (2019). Physical activity and your heart. https://www.nhlbi.nih.gov/health-topics/physical-activity-and-your-heart.

[6] (2019). Global physical activity surveillance. http://www.who.int/ncds/surveillance/steps/GPAQ/en.

[7] (2019). Monica manual, part III: population survey. https://thl.fi/publications/monica/manual/part3/iii-1.htm.

[8] WHO Expert Consultation (2004). Appropriate body mass index for Asian populations and its implications for policy and intervention strategies. Lancet.

[9] Alberti KGMM, Eckel RH, Grundy SM (2009). Harmonizing the metabolic syndrome: a joint interim statement of the International Diabetes Federation Task Force on epidemiology and prevention; National Heart, Lung, and Blood Institute; American Heart Association; World Heart Federation; International Atherosclerosis Society; and International Association for the Study of Obesity. Circulation.

[10] (2019). Global physical activity questionnaire (GPAQ) analysis guide. https://www.who.int/ncds/surveillance/steps/resources/GPAQ_Analysis_Guide.pdf.

[11] Bull FC, Maslin TS, Armstrong T (2009). Global physical activity questionnaire (GPAQ): nine country reliability and validity study. J Phys Act Health.

[12] Anjana RM, Pradeepa R, Das AK (2014). Physical activity and inactivity patterns in India - results from the ICMR-INDIAB study (phase 1) [ICMR-INDIAB-5]. Int J Behav Nutr Phys Act.

[13] Guthold R, Ono T, Strong KL, Chatterji S, Morabia A (2008). Worldwide variability in physical inactivity: a 51-country survey. Am J Prev Med.

[14] Macniven R, Bauman A, Abouzeid M (2012). A review of population-based prevalence studies of physical activity in adults in the Asia-Pacific region. BMC Public Health.

[15] Bauman A, Bull F, Chey T (2009). The International Prevalence Study on Physical Activity: results from 20 countries. Int J Behav Nutr Phys Act.

[16] Hallal PC, Andersen LB, Bull FC, Guthold R, Haskell W, Ekelund U (2012). Global physical activity levels: surveillance progress, pitfalls, and prospects. Lancet.

[17] Shah B, Mathur P (2010). Surveillance of cardiovascular disease risk factors in India: the need & scope. Indian J Med Res.

[18] Oommen AM, Abraham VJ, George K, Jose VJ (2016). Prevalence of risk factors for non-communicable diseases in rural & urban Tamil Nadu. Ind J Med Res.

[19] Bergman P, Grjibovski AM, Hagströmer M, Bauman A, Sjöström M (2008). Adherence to physical activity recommendations and the influence of socio-demographic correlates - a population-based cross-sectional study. BMC Public Health.

[20] Kinra S, Bowen LJ, Lyngdoh T (2010). Sociodemographic patterning of non-communicable disease risk factors in rural India: a cross sectional study. BMJ.

[21] World Health Organization (2014). Global Status Report on Noncommunicable Diseases 2014. diseases.

[22] Vaidya A, Krettek A (2014). Physical activity level and its sociodemographic correlates in a peri-urban Nepalese population: a cross-sectional study from the Jhaukhel-Duwakot health demographic surveillance site. Int J Behav Nutr Phys Act.

[23] Teh CH, Lim KK, Chan YY (2014). The prevalence of physical activity and its associated factors among Malaysian adults: findings from the National Health and Morbidity Survey 2011. Public Health.

[24] Nooyi SC, Sreekantaiah P, Shivananjaiah S, Rajaram D, Ugraiah K, Gowda V, Murthy NS (2016). Body Mass Index and its association with selected risk factors for non-communicable diseases in a rural area in Karnataka, India. Nat J Commun Med.

[25] Gornale VK, Sathenahalli VB, Kuber M, Singh HP (2015). A study of effect of physical activity quotient on central obesity (waist hip ratio) in adolescent girls and boys of central India. Int J Contemp Paed.

[26] Cheng LA, Mendonca G, Farias Júnior JC (2014). Physical activity in adolescents: analysis of the social influence of parents and friends. J Pediatr (Rio J).

[27] Hemmingsson E, Ekelund U (2007). Is the association between physical activity and body mass index obesity dependent?. Int J Obes.

[28] National Program for prevention and control of cancer, diabetes diabetes, CVD and stroke (NPCDCS (2019). National program for prevention and control of cancer, diabetes, CVD and stroke (NPCDCS). https://mohfw.gov.in/sites/default/files/7788412958a1-10837794.pdf.

[29] Laine J, Kuvaja-Köllner V, Pietilä E, Koivuneva M, Valtonen H, Kankaanpää E (2014). Cost-effectiveness of population-level physical activity interventions: a systematic review. Am J Health Promot.

